# Supplementary material for: Conformational disorder of organic cations tunes the charge carrier mobility in two-dimensional organic-inorganic perovskites
Source: Nat Commun. 2020 Oct 30;11:5481. doi: 10.1038/s41467-020-19330-7 (PMC7603336; doi:10.1038/s41467-020-19330-7)
Supplement: Supplementary file 1 — Supplementary Information [file 41467_2020_19330_MOESM1_ESM.pdf]

Supplementary Information

Conformational Disorder of Organic Cations Tunes the Charge Carrier Mobility in Two-dimensional Organic-Inorganic Perovskites

Chuanzhao Li, Jin Yang, Fuhai Su, Junjun Tan, Yi Luo, Shuji Ye,*

Supplementary Figure 1. Transient Terahertz (THz) transmission response of other five 2D OIHP thin films photoexcited at 400 nm. Excitation fluences range from 16 to 73.6 μJ·cm^{-2}. Symbols represent experimental data while solid lines are fitting to the data using bi-exponential function. **a** BA_2PbI_4 (n=4), **b** OA_2PbI_4 (n=8), **c** DA_2PbI_4 (n=10), **d** DDA_2PbI_4 (n=12), **e** ODA_2PbI_4 (n=18).

Supplementary Figure 2. The lifetime (τ) as the function of pump fluences ranging from 16 to 73.6 μJ·cm^{-2}.**a** Fast component (τ_1), **b** Slow component (τ_2).

Supplementary Figure 3. Maximum of |ΔT/T| as a function of excitation fluences in six 2D OIHP thin films, which saturates at higher fluence (>45 μJ·cm^{-2}).

Supplementary Figure 4. Dark current-voltage (I–V) curves with structure of ITO/2D OIHP/Al. **a** BA_2PbI_4 (n=4), **b** HA_2PbI_4 (n=6), **c** OA_2PbI_4 (n=8), **d** DA_2PbI_4 (n=10), **e** DDA_2PbI_4 (n=12), and **f** ODA_2PbI_4 (n=18).

Supplementary Figure 5. The pictures of the devices with ITO/2D OIHP/Al structure. **a** BA_2PbI_4 (n=4), **b** HA_2PbI_4 (n=6), **c** OA_2PbI_4 (n=8), **d** DA_2PbI_4 (n=10), **e** DDA_2PbI_4 (n=12), and **f** ODA_2PbI_4 (n=18).

Supplementary Figure 6. Temperature-dependent fluorescence mapping of other three 2D OIHP thin films obtained by heating from 80K to 300K and excited at 400 nm. **a** DA_2PbI_4 (n=10), **b** DDA_2PbI_4 (n=12), and **c** ODA_2PbI_4 (n=18).

Supplementary Figure 7. Fluorescence spectra of six 2D OIHPs at given temperature 80K, 100K, 150K, 200K, 250K, and 300K. **a** BA_2PbI_4 (n=4), **b** HA_2PbI_4 (n=6), **c** OA_2PbI_4 (n=8), **d** DA_2PbI_4 (n=10), **e** DDA_2PbI_4 (n=12), and **f** ODA_2PbI_4 (n=18).

Supplementary Figure 8. Fluorescence spectra and their Gaussian fit of six 2D OIHPs at 80K. The black dots are the experimental data, red lines are fitted by multi-Gaussian functions,

narrow emission peak 1, 2 and 3 are abbreviated by NE1, NE2 and NE3, white-light broadband emission peak is abbreviated by BE. **a** BA_2PbI_4 (n=4), **b** HA_2PbI_4 (n=6), **c** OA_2PbI_4 (n=8), **d** DA_2PbI_4 (n=10), **e** DDA_2PbI_4 (n=12), and **f** ODA_2PbI_4 (n=18).

Supplementary Figure 9. The SFG results of the films with a thickness of 144 nm. **a** ppp spectra. **b** The measured ppp and ssp intensity ratio ($\chi^{(2)}_{ppp}/\chi^{(2)}_{ssp}$) of symmetric methyl group.

Supplementary Figure 10. The SFG results of the films with a thickness of 9.0 nm. **a** ssp spectra. **b** ppp spectra. **c** The $\chi^{(2)}_{\mathrm{ss-\ CH_3}}/\chi^{(2)}_{\mathrm{ss-\ CH_2}}$ ratio is plotted against the alkyl chain length of organic cations. **d** The measured ppp and ssp intensity ratio ($\chi^{(2)}_{\mathrm{ppp}}/\chi^{(2)}_{\mathrm{ssp}}$) of symmetric methyl group. **e** The in-plane mobility is correlated with the $\chi^{(2)}_{\mathrm{ss-\ CH_3}}/\chi^{(2)}_{\mathrm{ss-\ CH_2}}$ ratio.

Supplementary Figure 11. The SFG results of the films with a thickness of 55 nm. **a** ssp spectra. **b** ppp spectra. **c** The $\chi^{(2)}_{\mathrm{ss-\ CH_3}}/\chi^{(2)}_{\mathrm{ss-\ CH_2}}$ ratio is plotted against the alkyl chain length of organic cations. **d** The measured ppp and ssp intensity ratio ($\chi^{(2)}_{\mathrm{ppp}}/\chi^{(2)}_{\mathrm{ssp}}$) of symmetric methyl group. **e** The in-plane mobility is correlated with the $\chi^{(2)}_{\mathrm{ss-\ CH_3}}/\chi^{(2)}_{\mathrm{ss-\ CH_2}}$ ratio.

Supplementary Figure 12. The surface roughness and spatial homogeneity of the films determined by AFM measurements. The roughness is 2.0±1.0 nm for the films of n=4-8 and 10.0±2.0 nm for the films of n=10-18. The correlation between the alkyl chain conformation and the roughness is not observed.

Supplementary Figure 13. The simulated XRD patterns of six 2D OIHPs using VESTA.

Supplementary Figure 14. Optical layout of the employed optical pump-THz probe (OPTP) system. BBO: Beta-Barium Borate crystal, BS: beam splitter, DS: delay stage, OC: optical chopper, PD: photodetector, PM: parabolic mirror, QWP: quarter wave plate, WP: Wollaston prism.

Supplementary Figure 15. The transmission and reflection spectra of six 2D OIHP thin films measured by UV-Vis-IR spectrophotometer at normal incidence of the UV-Vis beam. **a** transmittance (%) and **b** reflectivity (%).

SupplementaryTable 1. The examples for the charge-carrier mobility values determined by OPTPS measurement.

Supplementary Table 2. The trap density and charge mobility derived from Supplementary Figure 4.

Supplementary Table 3. The comparison of the value of (002) peaks in 2D OIHP films given by experiment and VESTA program.

Supplementary Table 4. The transmittance and reflectivity of six 2D OIHP thin films at normal incidence of 400 nm.

Supplementary Note 1. Calculation of photoconductivity and charge carrier mobility from the change in peak THz amplitude (ΔT/T)

Supplementary Note 2. Fitting of THz transmission response of the 2D OIHP thin films.

Supplementary Note 3. The lifetime (τ) as a function of pump fluences.

Supplementary Note 4. Estimation of out-of-plane mobility and trap density by Mott−Gurney analysis of the I−V data curves.

Supplementary Note 5. Fluorescence spectra at 80K and their Gaussian fit of six 2D OIHPs.

Supplementary Note 6. Spectral fitting of SFG-VS signals.

Supplementary Note 7. The possible influence of chain orientation and film thickness on the $\chi^{(2)}_{ss-CH_3}/\chi^{(2)}_{ss-CH_2}$ ratio.

Supplementary Note 8. Layout of the employed optical pump-terahertz probe (OPTP) system.

Supplementary Note 9. The XRD patterns of six 2D OIHP thin films.

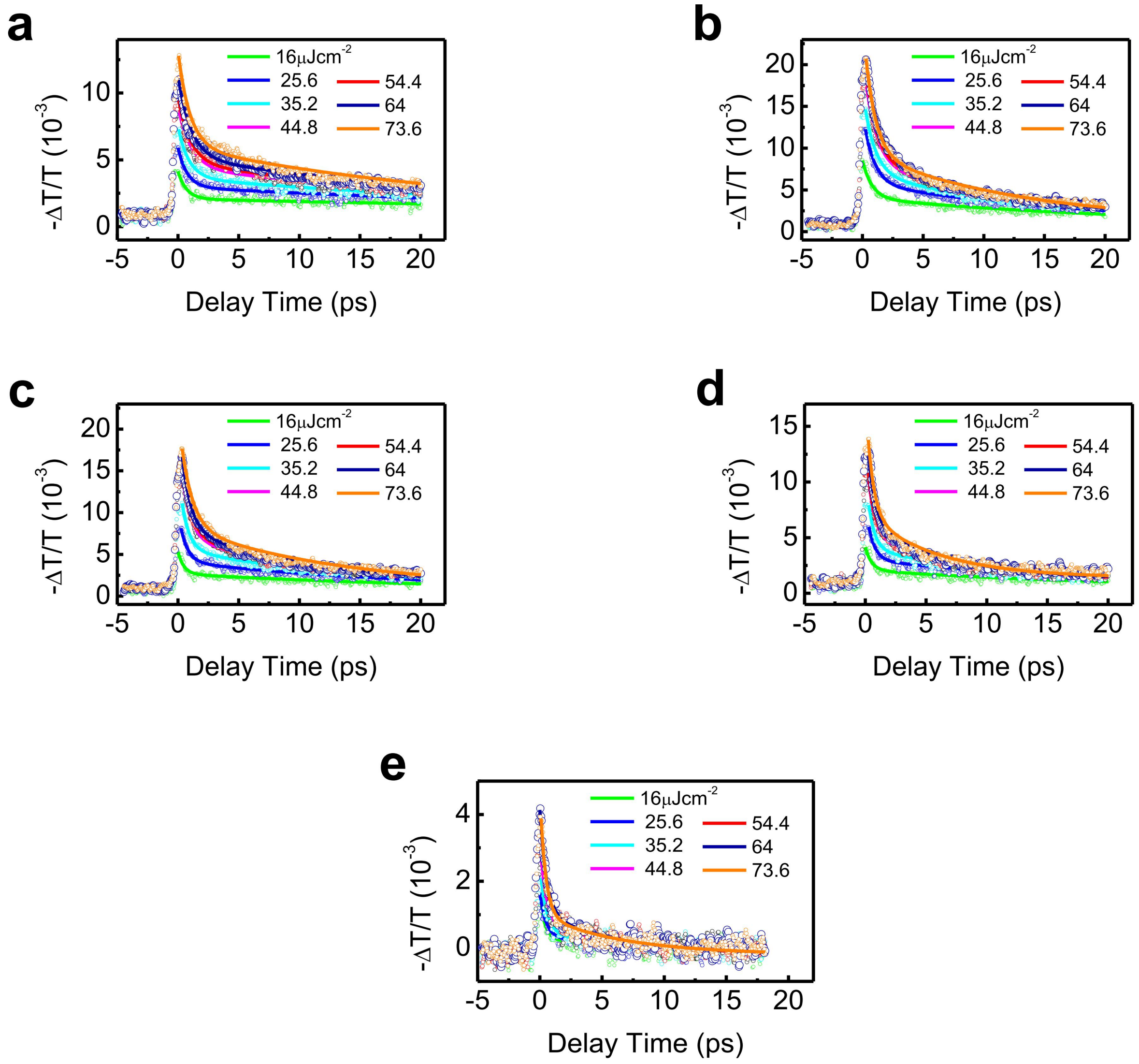

Supplementary Figure 1. Transient Terahertz (THz) transmission response of other five 2D OIHP thin films photoexcited at 400 nm. Excitation fluences range from 16 to 73.6 μJ·cm^{-2}. Symbols represent experimental data while solid lines are fitting to the data using bi-exponential function. **a** BA_2PbI_4 (n=4), **b** OA_2PbI_4 (n=8), **c** DA_2PbI_4 (n=10), **d** DDA_2PbI_4 (n=12), **e** ODA_2PbI_4 (n=18).

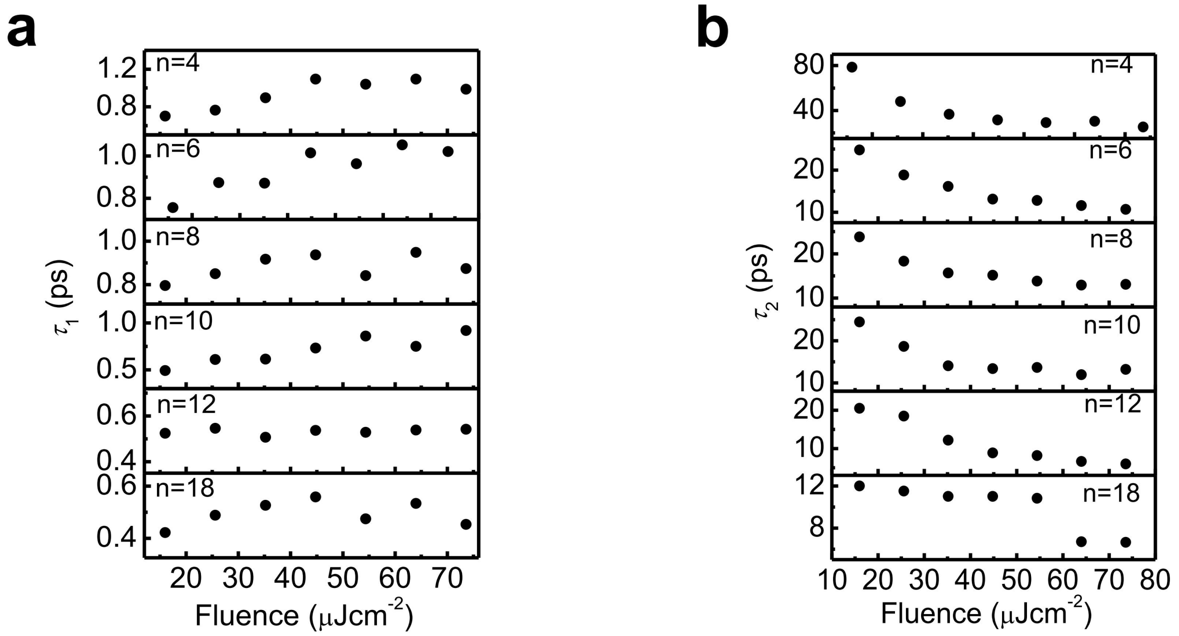

Supplementary Figure 2. The lifetime (τ) as the function of pump fluences ranging from 16 to 73.6 μJ·cm^{-2}. a Fast component (τ_1), **b** Slow component (τ_2).

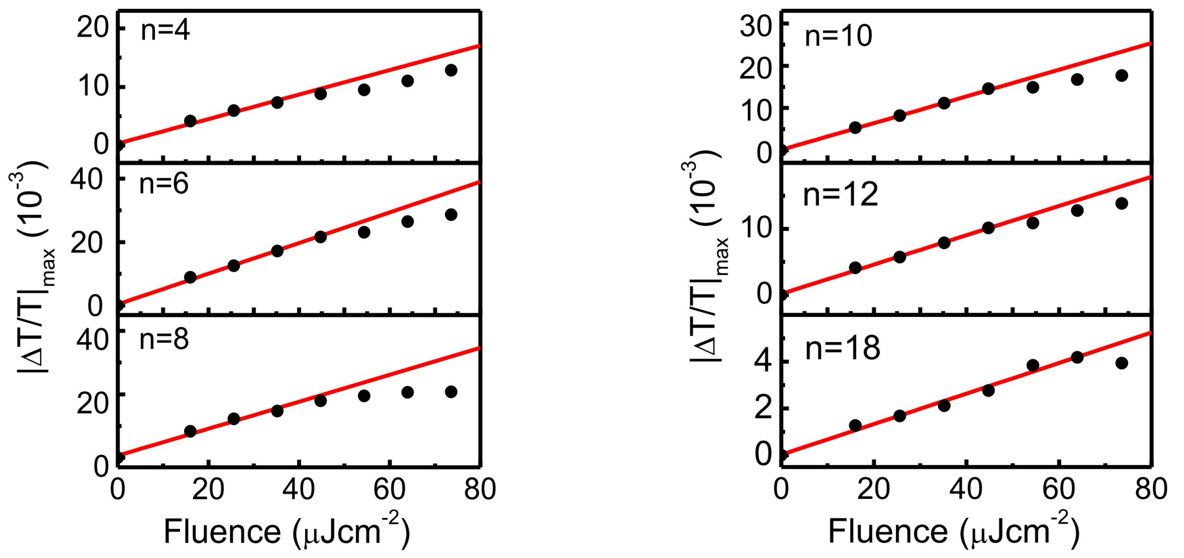

Supplementary Figure 3. Maximum of |ΔT/T| as a function of excitation fluences in six 2D OIHP thin films, which saturates at higher fluence (>45 μJ·cm^{-2}).

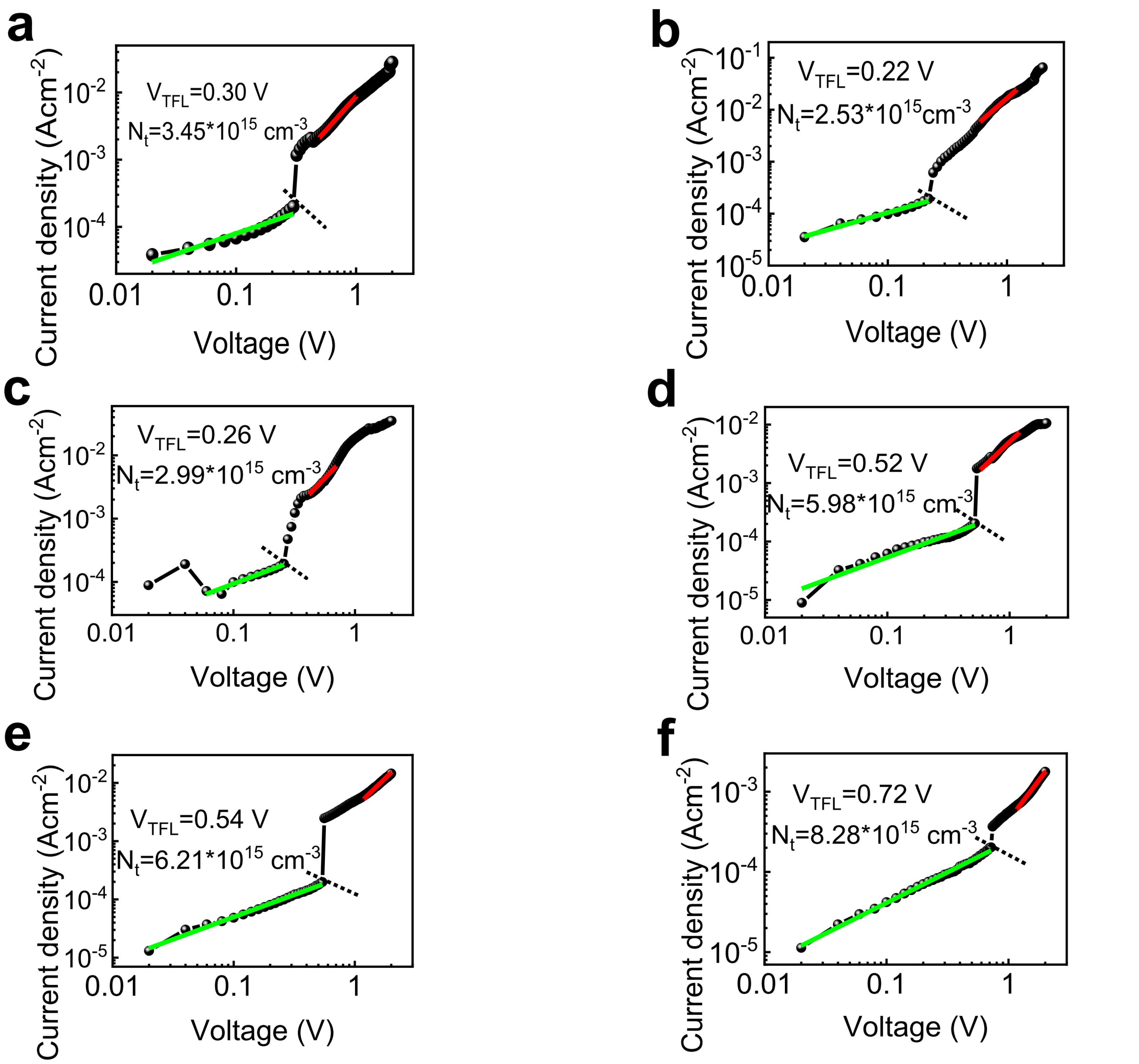

Supplementary Figure 4. Dark current-voltage (I–V) curves with structure of ITO/2D OIHP/Al. **a** BA_2PbI_4 (n=4), **b** HA_2PbI_4 (n=6), **c** OA_2PbI_4 (n=8), **d** DA_2PbI_4 (n=10), **e** DDA_2PbI_4 (n=12), and **f** ODA_2PbI_4 (n=18).

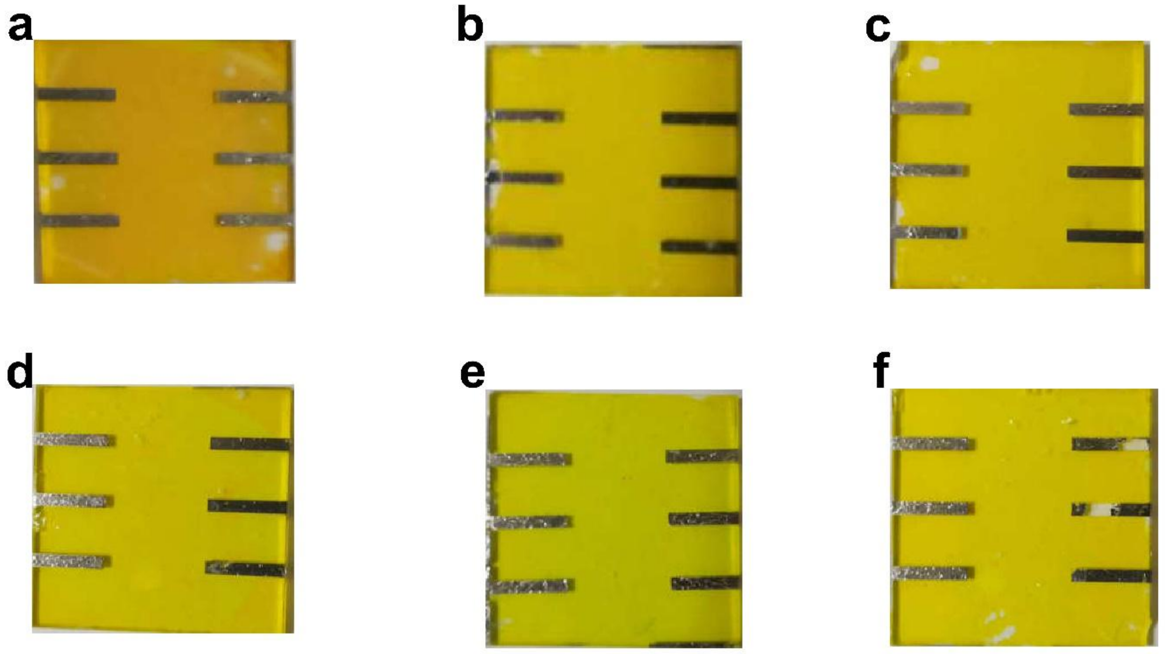

Supplementary Figure 5. The pictures of the devices with ITO/2D OIHP/Al structure.

a BA_2PbI_4 (n=4), **b** HA_2PbI_4 (n=6), **c** OA_2PbI_4 (n=8), **d** DA_2PbI_4 (n=10), **e** DDA_2PbI_4 (n=12), and **f** ODA_2PbI_4 (n=18).

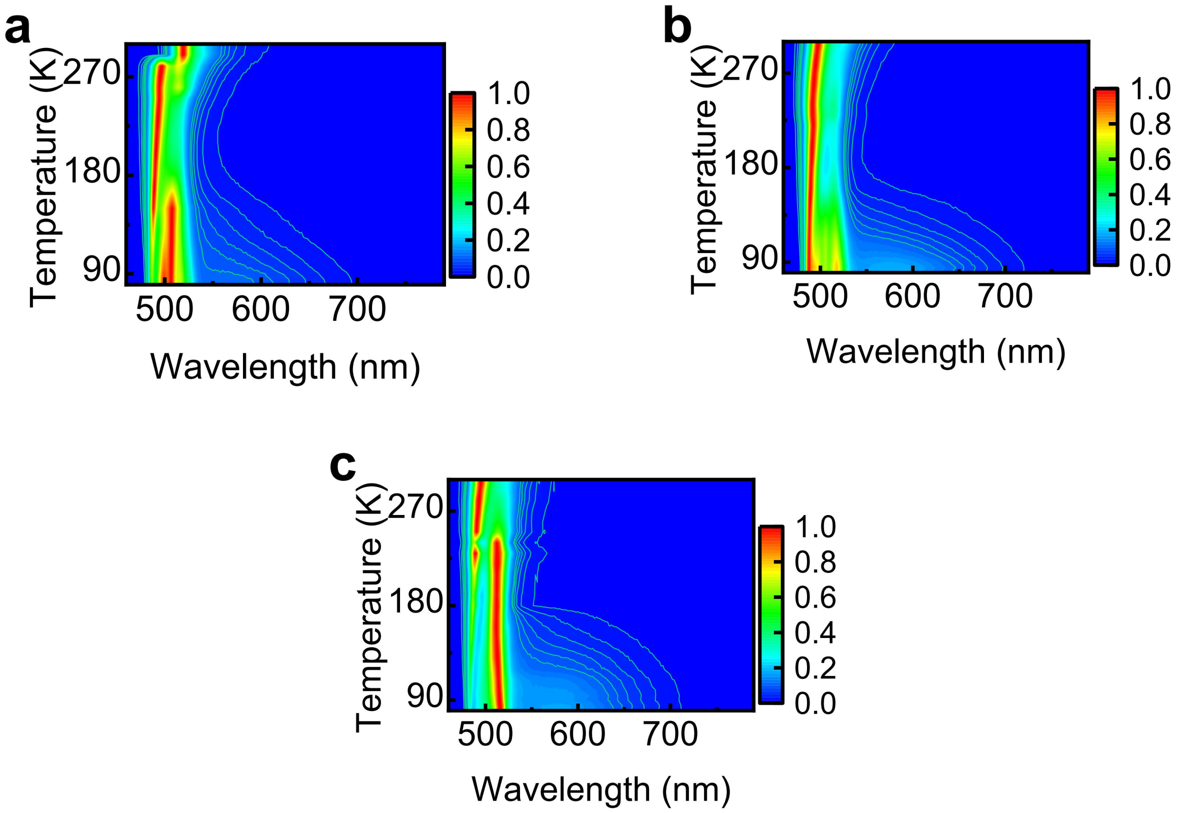

Supplementary Figure 6. Temperature-dependent fluorescence mapping of other three 2D OIHP thin films obtained by heating from 80K to 300K and excited at 400 nm. a DA_2PbI_4 (n=10), **b** DDA_2PbI_4 (n=12), and **c** ODA_2PbI_4 (n=18).

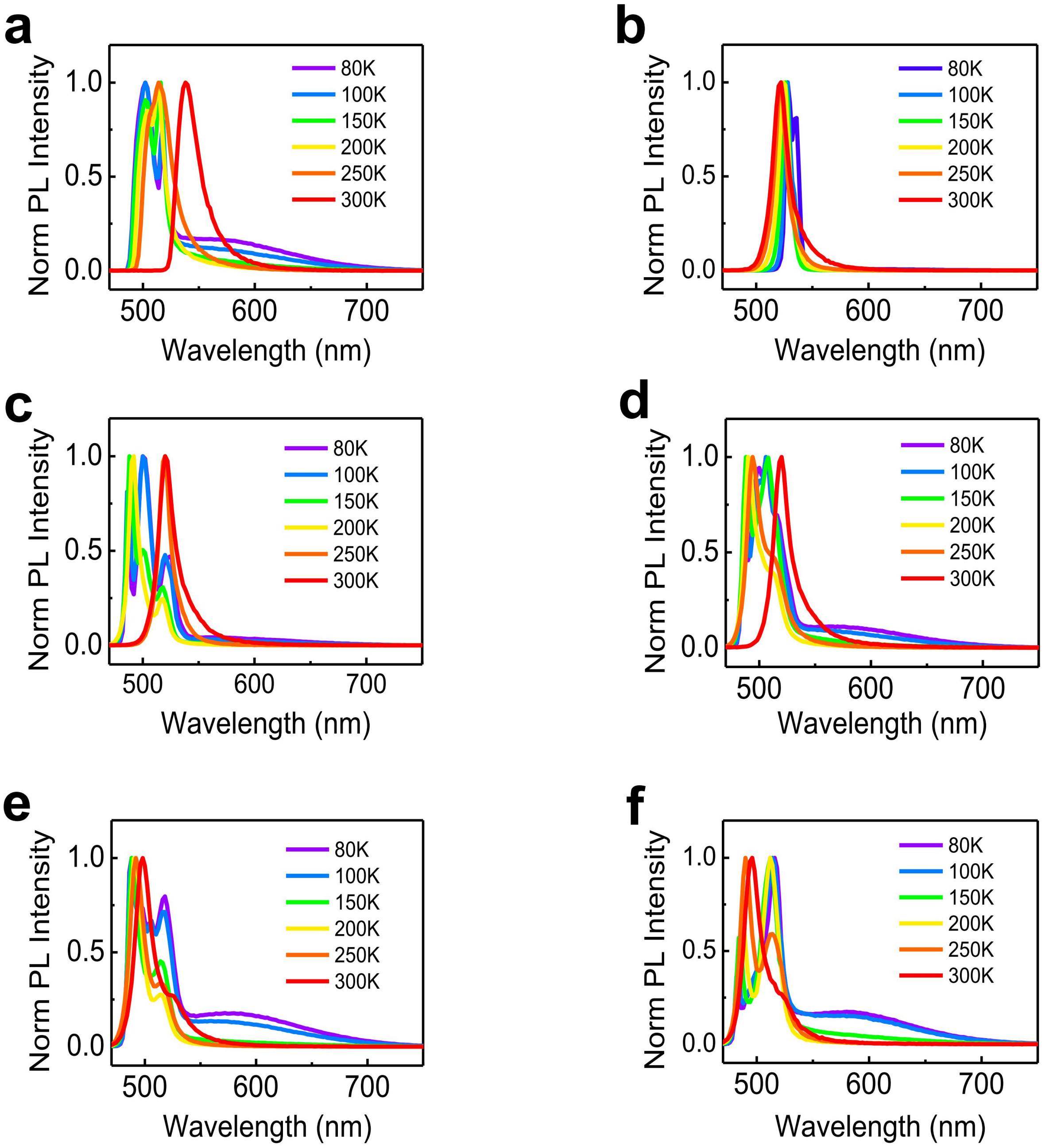

Supplementary Figure 7. Fluorescence spectra of six 2D OIHPs at given temperature 80K, 100K, 150K, 200K, 250K, and 300K. a BA_2PbI_4 (n=4), **b** HA_2PbI_4 (n=6), **c** OA_2PbI_4 (n=8), **d** DA_2PbI_4 (n=10), **e** DDA_2PbI_4 (n=12), and **f** ODA_2PbI_4 (n=18).

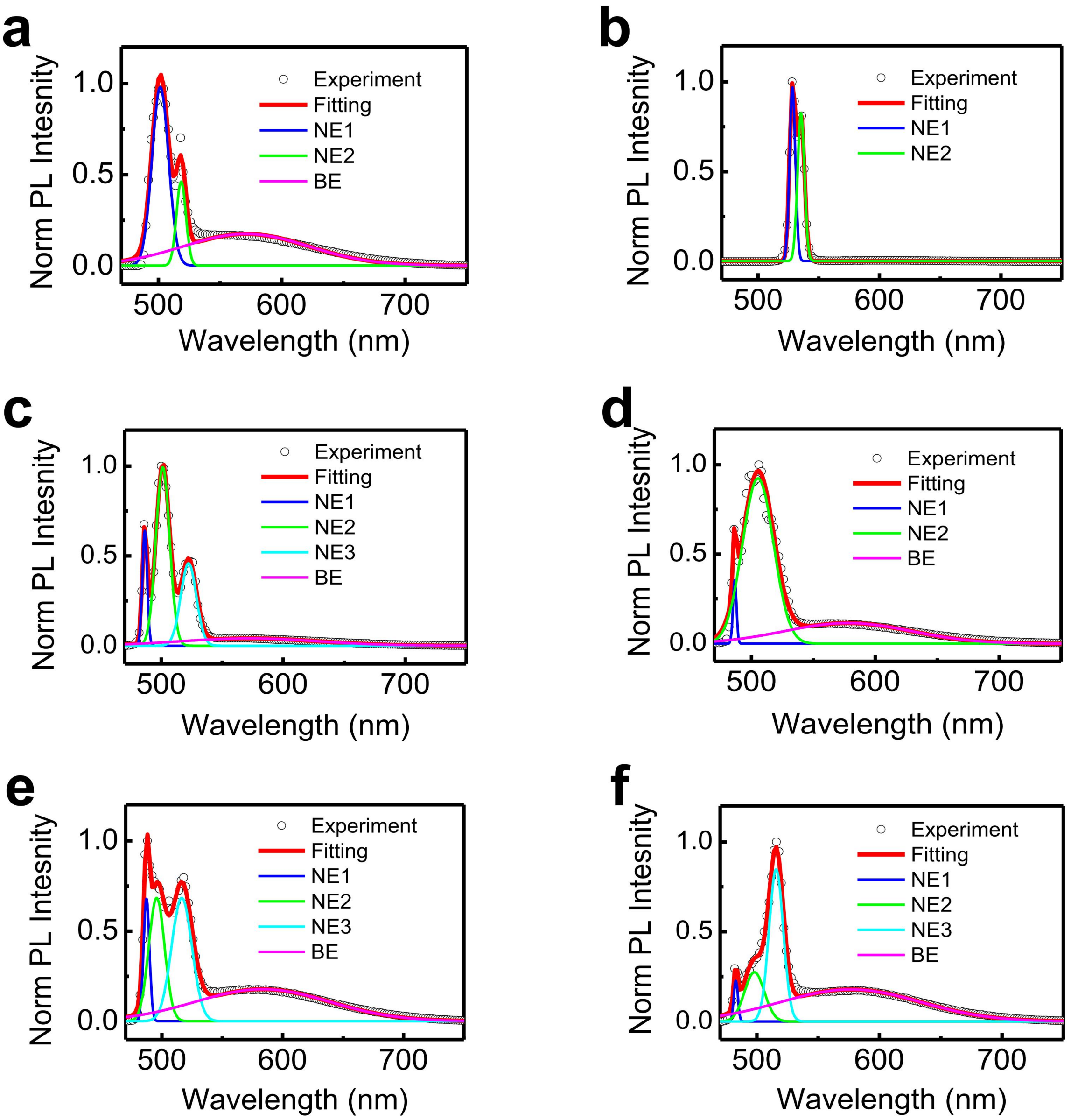

Supplementary Figure 8. Fluorescence spectra and their Gaussian fit of six 2D OIHPs at 80K. The black dots are the experimental data, red lines are fitted by multi-Gaussian functions, narrow emission peak 1, 2 and 3 are abbreviated by NE1, NE2 and NE3, white-light broadband emission peak is abbreviated by BE. **a** BA_2PbI_4 (n=4), **b** HA_2PbI_4 (n=6), **c** OA_2PbI_4 (n=8), **d** DA_2PbI_4 (n=10), **e** DDA_2PbI_4 (n=12), and **f** ODA_2PbI_4 (n=18).

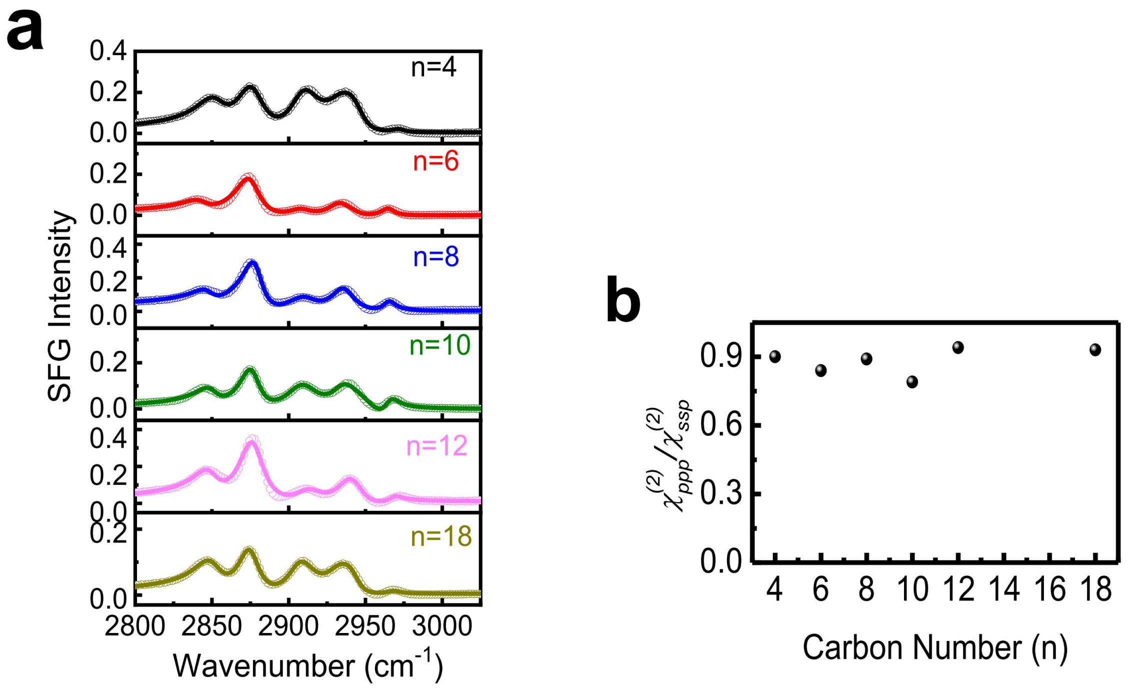

Supplementary Figure 9. The SFG results of the films with a thickness of 144 nm. a ppp spectra. **b** The measured ppp and ssp intensity ratio ($\chi^{(2)}_{ppp}/\chi^{(2)}_{ssp}$) of symmetric methyl group.

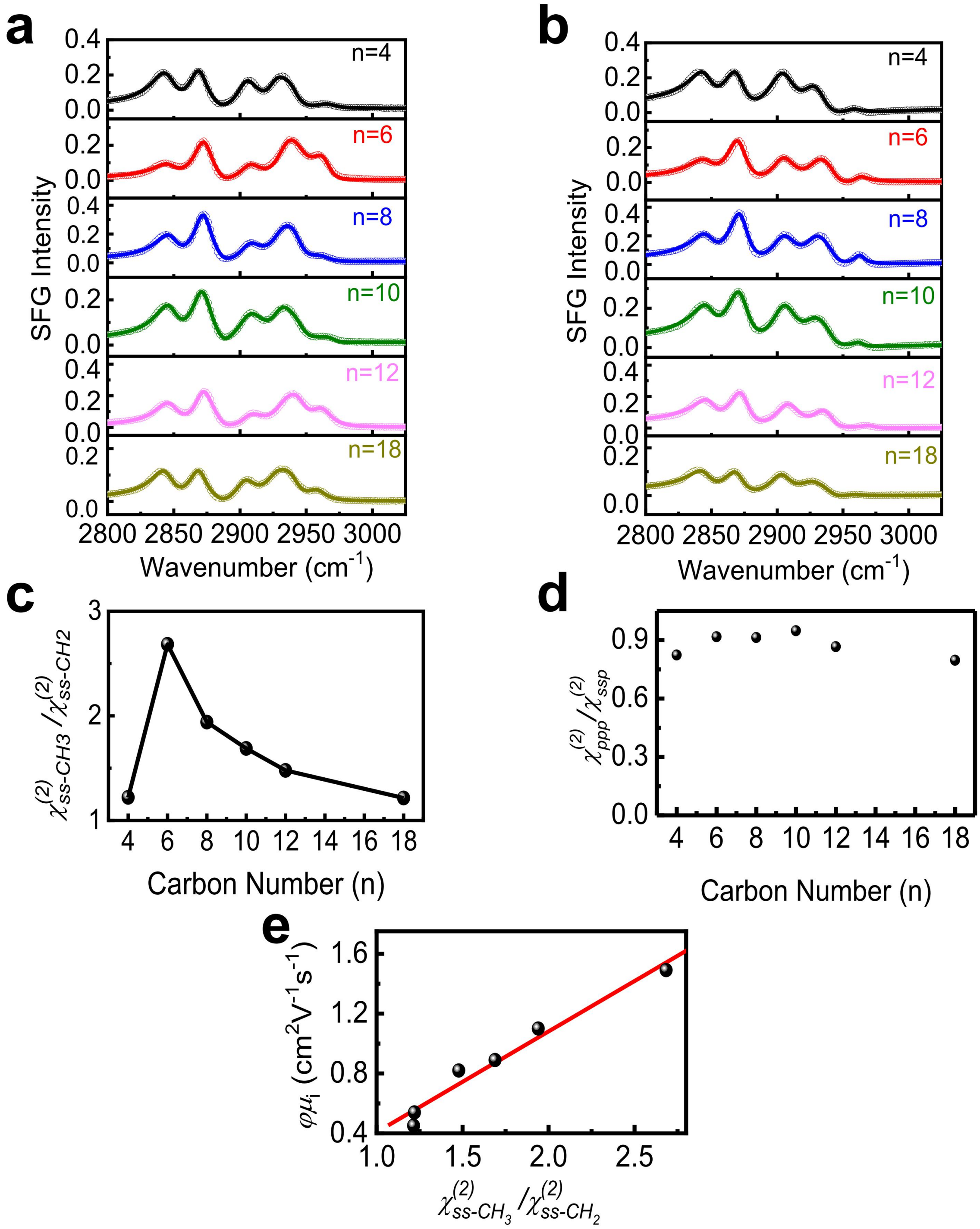

Supplementary Figure 10. The SFG results of the films with a thickness of 9.0 nm. a ssp spectra. **b** ppp spectra. **c** The $\chi^{(2)}_{ss-\mathrm{CH_3}}/\chi^{(2)}_{ss-\mathrm{CH_2}}$ ratio is plotted against the alkyl chain length of organic cations. **d** The measured ppp and ssp intensity ratio ($\chi^{(2)}_{ppp}/\chi^{(2)}_{ssp}$) of symmetric methyl group. **e** The in-plane mobility is correlated with the $\chi^{(2)}_{ss-\mathrm{CH_3}}/\chi^{(2)}_{ss-\mathrm{CH_2}}$ ratio.

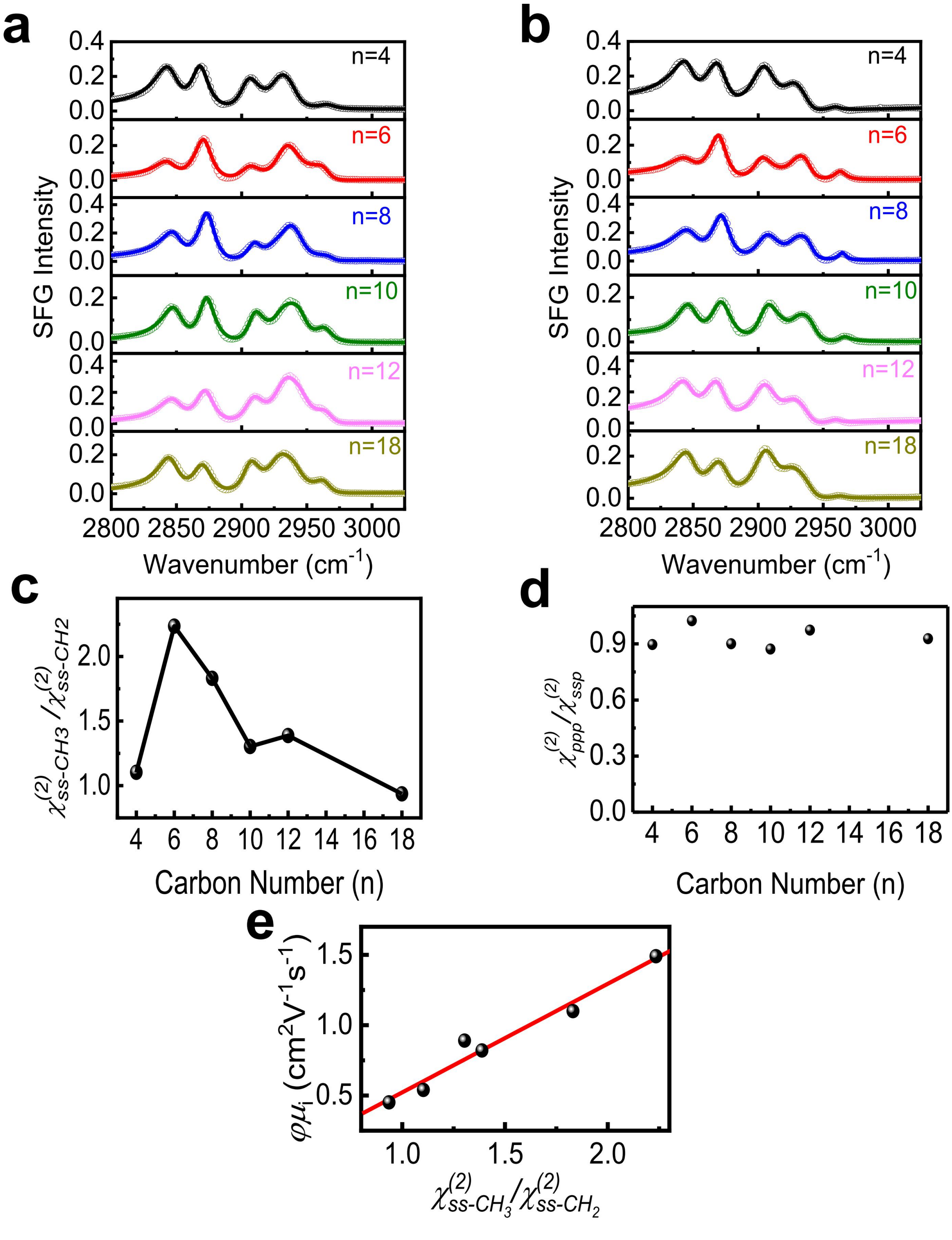

Supplementary Figure 11. The SFG results of the films with a thickness of 55 nm. **a** ssp spectra. **b** ppp spectra. **c** The $\chi^{(2)}_{ss-CH_3}/\chi^{(2)}_{ss-CH_2}$ ratio is plotted against the alkyl chain length of organic cations. **d** The measured ppp and ssp intensity ratio ($\chi^{(2)}_{ppp}/\chi^{(2)}_{ssp}$) of symmetric methyl group. **e** The in-plane mobility is correlated with the $\chi^{(2)}_{ss-CH_3}/\chi^{(2)}_{ss-CH_2}$ ratio.

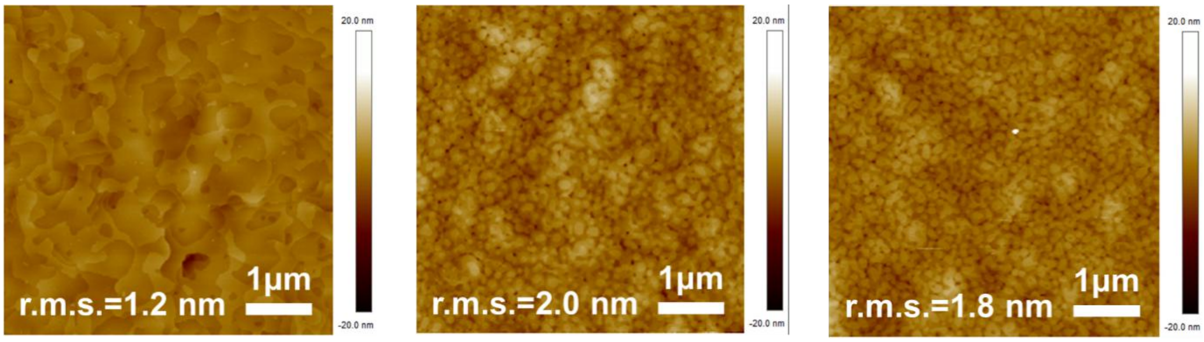

BA (n=4) HA (n=6) OA (n=8)

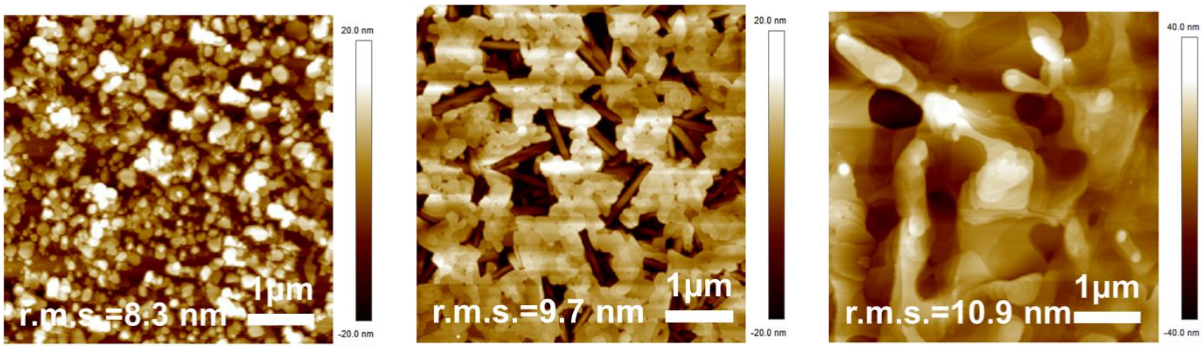

DA (n=10) DDA (n=12) ODA (n=18)

Supplementary Figure 12. The surface roughness and spatial homogeneity of the films determined by AFM measurements. The roughness is 2.0±1.0 nm for the films of n=4-8 and 10.0±2.0 nm for the films of n=10-18. The correlation between the alkyl chain conformation and the roughness is not observed.

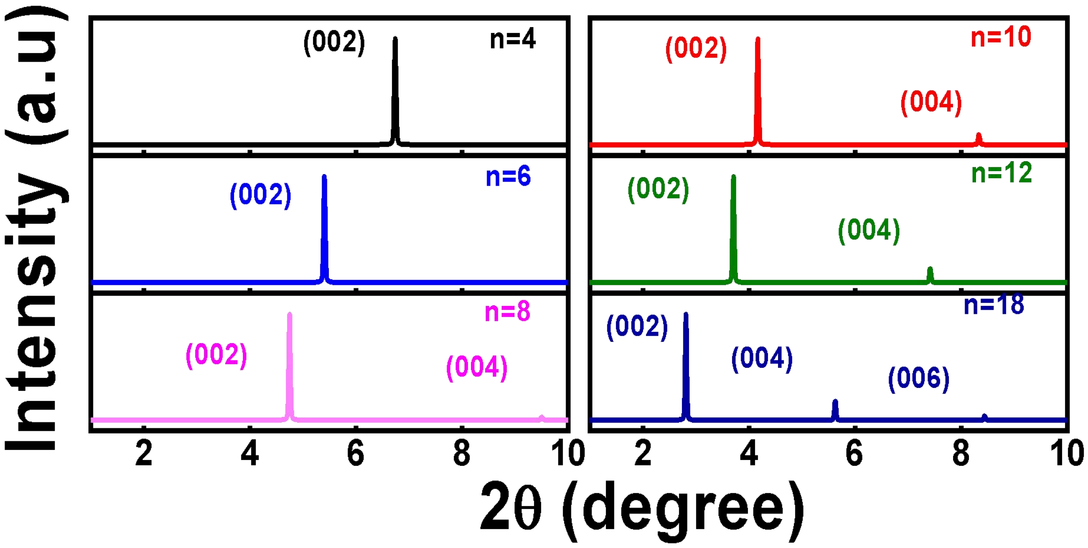

Supplementary Figure 13. The simulated XRD patterns of six 2D OIHPs using VESTA.

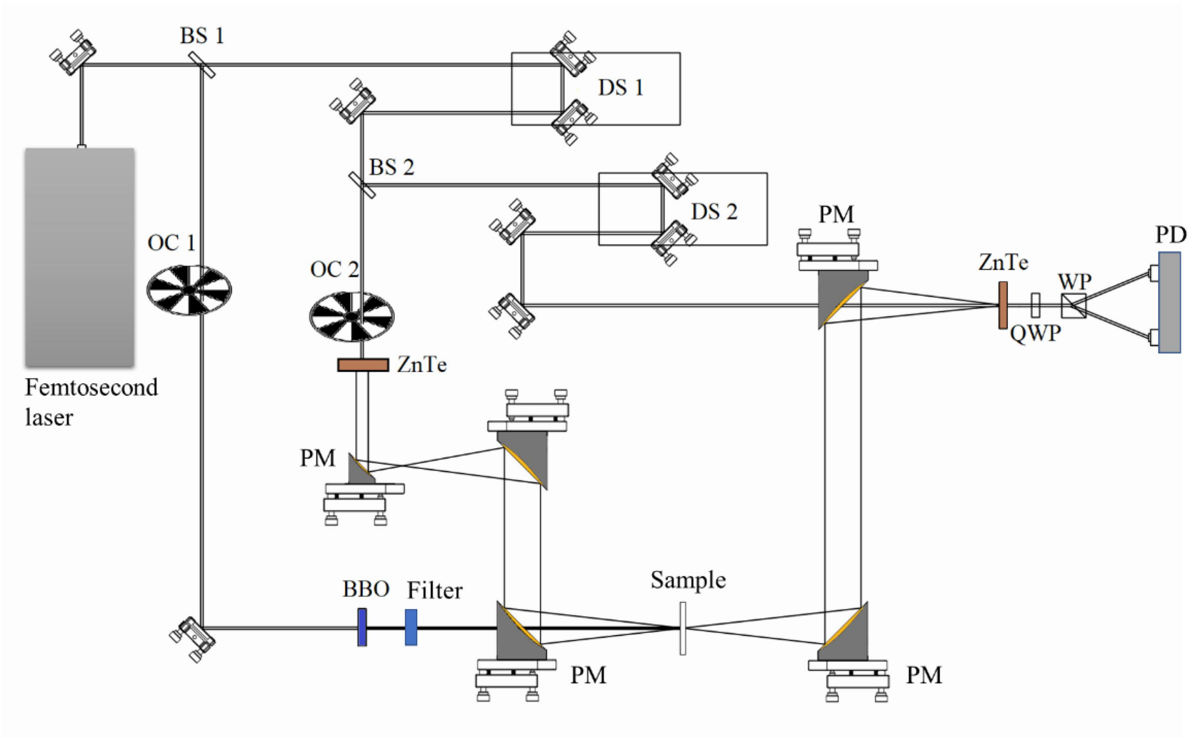

Supplementary Figure 14. Optical layout of the employed optical pump-THz probe (OPTP) system. BBO: Beta-Barium Borate crystal, BS: beam splitter, DS: delay stage, OC: optical chopper, PD: photodetector, PM: parabolic mirror, QWP: quarter wave plate, WP: Wollaston prism.

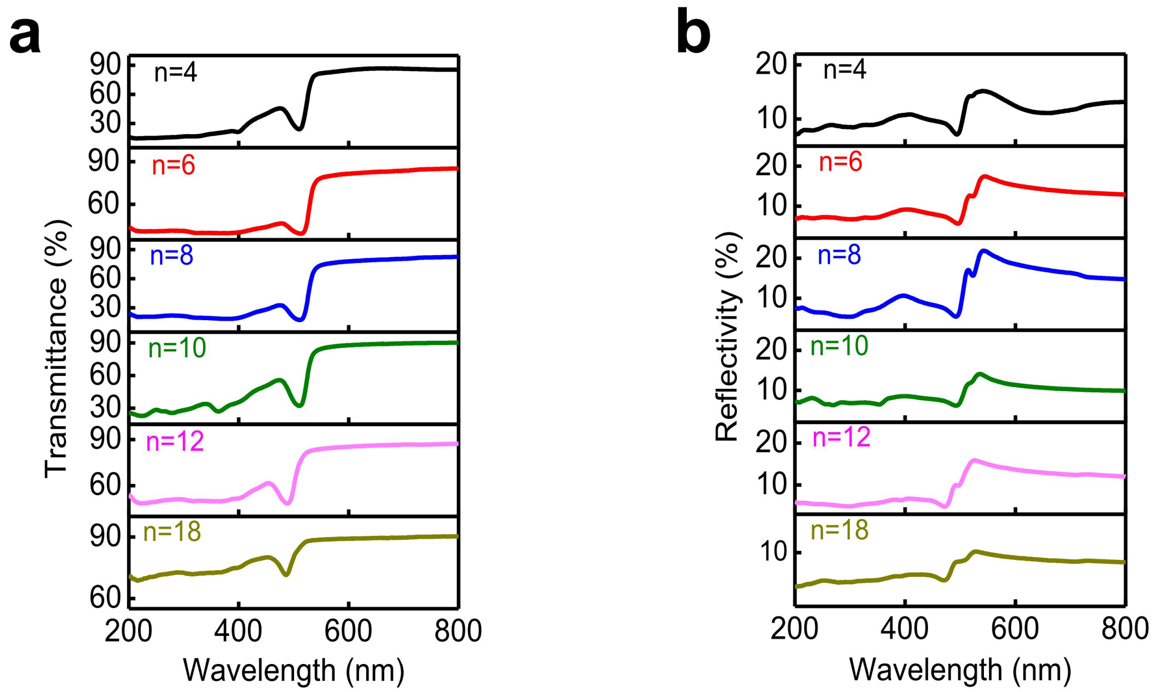

Supplementary Figure 15. The transmission and reflection spectra of six 2D OIHP thin films measured by UV-Vis-IR spectrophotometer at normal incidence of the UV-Vis beam. a transmittance (%) and **b** reflectivity (%).

Supplementary Table 1. The examples for the charge-carrier mobility values determined by OPTPS measurement.

Composition	Architecture	Mobility ($cm^2 \cdot V^{-1} \cdot s^{-1}$)	Reference
$MAPbI_3$	Films	35	1
$MAPbI_3$	Films	20	2
$MAPbI_3$	Single Crystal	600	3
$MAPbI_{3-x}ICl_x$	Films	33	4
$MAPbI_{3-x}ICl_x$	Films	27	5
$FAPb(Br_xI_{1-x})_3(0 \leq x \leq 1)$	Films	1-27	6
$Cs_{0.17}FA_{0.83}PbI_3$	Films	40	7
$Cs_yFA_{1-y}Pb(Br_{0.4}I_{0.6})_3$	Films	4-21	7，8
$Cs_{0.17}FA_{0.83}Pb(Br_xI_{1-x})_3$	Films	11-40	7，9
$MASnI_3$	Mesoporous matrix	1.6	10
$FASnI_3$	Film	22	11
$FASn_{0.5}Pb_{0.5}I_3$	Films	17	12
$Cs_{0.25}FA_{0.75}Sn_{0.5}Pb_{0.5}I_3$	Films	14	12
$MA_{n-1}PEA_2Pb_nI_{3n+1}$	Films (2D)	1-25	13
$BA_x(FA_{0.83}Cs_{0.17})_{1-x}Pb(I_{0.6}Br_{0.4})_3$ $(x \leq 0.8)$	Films	3-15	14
$CsPbI_3$	Quantum dots	0.23-0.5	15
PbS	Quantum dots	0.042	15
PbSe	Quantum dots	0.09	15
$FAPb(Br_{0.3}I_{0.7})_3$	Films	2	6
$FAPb(Br_{0.4}I_{0.6})_3$	Films	1	6

Supplementary Table 2. The trap density and charge mobility derived from Supplementary Figure 4.

Samples	N_t(10^{15} cm^{-3})	Mobility(10^{-4} $cm^2 \cdot V^{-1} \cdot s^{-1}$)
BA_2PbI_4 (n=4)	3.45	1.4
HA_2PbI_4 (n=6)	2.53	2.7
OA_2PbI_4 (n=8)	2.99	2.2
DA_2PbI_4 (n=10)	5.98	0.83
DDA_2PbI_4 (n=12)	6.21	0.61
ODA_2PbI_4 (n=18)	8.28	0.072

Supplementary Table 3. The comparison of the value of (002) peaks in 2D OIHP films given by experiment and VESTA program.

Samples	Experiment (°)	VESTA (°)	Relative error (%)
BA_2PbI_4 (n=4)	6.49	6.74	3.71
HA_2PbI_4 (n=6)	5.34	5.40	1.11
OA_2PbI_4 (n=8)	4.84	4.75	1.89
DA_2PbI_4 (n=10)	4.26	4.17	2.16
DDA_2PbI_4 (n=12)	3.71	3.71	0.00
ODA_2PbI_4 (n=18)	2.86	2.81	1.78

Supplementary Table 4. The transmittance and reflectivity of six 2D OIHP thin films at normal incidence of 400 nm.

Samples	Transmittance (%)	Reflectivity (%)
BA_2PbI_4 (n=4)	21.40	10.77
HA_2PbI_4 (n=6)	40.24	9.13
OA_2PbI_4 (n=8)	19.65	10.59
DA_2PbI_4 (n=10)	35.44	8.53
DDA_2PbI_4 (n=12)	51.68	6.59
ODA_2PbI_4 (n=18)	75.07	7.04

Supplementary Note 1. Calculation of photoconductivity and charge carrier mobility from the change in peak THz amplitude (ΔT/T)

When the thickness of film is much smaller than the THz wavelength, the sheet photoconductivity of the 2D OIHP thin film between two media with the refractive index, n_A and n_B, can be expressed by SupplementaryEq. 1[13,16].

$$\Delta S = -\varepsilon_0 c(n_A + n_B)(\Delta T/T), \quad (1)$$

where ε_0 is the permittivity of vacuum,c is the light speed, ΔT/T is the experimentally determined change in transmitted THz electric field amplitude. In our experiments, the 2D OIHP thin film is surrounded by dry nitrogen from one side and the z-cut quartz substrate with THz-refractive index (n= 2.13) from other side, hence n_A=1, n_B = 2.13.

The number of photoexcited charge carriers N can be derived by Supplementary Eq. 2.

$$N = \varphi \frac{E\lambda}{hc}(1 - R_{pump})(1 - T_{pump}), \quad (2)$$

Here, E is the energy contained in an optical excitation pulse of wavelengthλ, in this experiment, λ=400 nm,his Planck constant, R_{pump} and T_{pump}represent the reflectivity and the transmittance of the sample at normal incidence of the excitation beam, respectively.φis the photon-to-charge branching ratio, which is closely related to the dimensionality of perovskites. According to previous publications[13,17], in 3D perovskites, the exciton features can be negligible at room temperature, so φ=1. However, due to large exciton binding energy (~320 meV) [13,18], we cannot assume that φ=1 for 2D OIHPs.

The charge carrier is given by the following equation:

$$\mu = \frac{\Delta S A_{eff}}{Ne} \quad (3)$$

Here, A_{eff} is the effective overlap area of the optical pump and THz probe pulse, e is the elementary charge. Substituting Supplementary Eq. 1 and Supplementary Eq. 2 into Supplementary Eq. 3, thus Supplementary Eq. 4 can be obtained:

$$\varphi\mu = -\varepsilon_0 c(n_A + n_B)\frac{A_{eff}hc}{Ee\lambda(1-R_{pump})(1-T_{pump})}(\Delta T/T) \quad (4)$$

Due to $0 \leq \varphi \leq 1$, the effective charge carrier mobility is a lower limit, which is the same as the actual mobility for all photon to free carrier conversion. It is worth noting that the charge carrier mobility in this experiment is contributed from both electrons and holes and these contributions cannot be separated. Therefore, the extracted charge carrier mobility value

is the sum of electron and hole mobility.

To accurately determine the value of $\varphi\mu$, we must ensure that the excitation conditions are in the linear regime. We have evaluated the change in the peak of THz amplitude (ΔT) as a function of excitation fluences, and found that the nonlinear process such as two-photon absorption or hot phonon nesting effects had appeared when the pump fluence is larger than 44.8 $\mu J\cdot cm^{-2}$ (see Supplementary Figure 2). Our effective charge carrier mobility calculation is based on data when the pump fluence is 25.6 $\mu J\cdot cm^{-2}$ and therefore is not affected.

In addition, to obtain the value of $\varphi\mu$, we should know R_{pump} and T_{pump}. Therefore, we measured the transmission and reflection spectra (see Supplementary Figure 15) using a SolidSpec-3700 UV-vis-NIR spectrophotometer (Shimadzu Corporation, Japan) with blank substrate correction. The spectra were collected with a wavelength range of 200 nm ≤ λ≤ 800 nm and Δλ= 1nm.The deduced value of R_{pump} and T_{pump} is shown in Supplementary Table 4.

Supplementary Note 2. Fitting of THz transmission response of the 2D OIHP thin films

Supplementary Figure 1 shows THz transmission response of the 2D OIHP thin films (BA_2PbI_4, OA_2PbI_4, DA_2PbI_4, DDA_2PbI_4, ODA_2PbI_4) photoexcited at 400 nm with the pump fluences ranging from 16 to 73.6 $\mu J\cdot cm^{-2}$.In Supplementary Figure 1, symbols represent experimental data while solid lines are fitting curve using bi-exponential function (Supplementary Eq. 5) [15,19,20].

$$y = y_0 + A_1 e^{-x/T_1} + A_2 e^{-x/T_2} \quad (5)$$

Supplementary Note 3. The lifetime (τ) as a function of pump fluences

Supplementary Figure 2 shows the lifetime (τ) of six 2D OIHP films with the pump fluences ranging from 16 to 73.6 $\mu J\cdot cm^{-2}$. It is found that the fast component τ_1 is independent on the pump fluence and its timescale is about 1 ps. This fast process is attributed to the electron-phonon scattering process according to the conclusions of former publications[4-6]. The component τ_2 decreases as the pump fluence increases,indicating that it is dominated by a many-body interaction,such as the Auger process[21-23].

Supplementary Note 4. Estimation of out-of-plane mobility and trap density by Mott−Gurney analysis of the I−V data curves

Devices used for the Mott-Gurney current-voltage (I-V) measurements were fabricated with an ITO/Perovskite/Al structure (Supplementary Figure 5). The perovskite layer was spin-coated on the ITO glass with the standard procedure mentioned in the main text. Al layers were sequentially evaporated on the top of the perovskite films. The perovskite thickness of these devices was ~144 nm. The device area was 1 mm^2 as defined by the overlapping area of the ITO films and top Al electrodes. Finally, devices were tested in ambient air with a Keithley 2400 source meter with a voltage ramp rate of $0.02 V \cdot s^{-1}$.

In terms of Mott-Gurney (M-G) analysis, the dark current-voltage (I-V) curves were used to evaluate the trap density and charge mobility of devices with the structure of ITO/2D OIHP/Al (Supplementary Figure 5). As shown in the Supplementary Figure 7, the I-V curves with double-logarithmic scale under dark condition show three typical fitting straight lines regions: a linear ohmic region at low bias (slope k = 1) (green line), a trap-filled region at intermediate bias region(slope k >3), and a trap-free space charge limited current (SCLC) region at high bias (slope k = 2) (red line). The bias voltage at the kink point between the ohmic region and the trap-filled region is defined as trap-filled limited voltage (V_{TFL}). The charge trap-state density (N_t) can be determined by the trap-filled limit voltage (V_{TFL}) using Supplementary Eq. 6[17, 24]

$$N_t = \frac{2\varepsilon_0 \varepsilon_r V_{TFL}}{qd^2} \qquad (6)$$

where ε_0, ε_r, q and d is the vacuum permittivity (8.85×10^{-12} $F\cdot m^{-1}$), the relative dielectric constant (2.07), elementary charge (1.6×10^{-19}C), and the thickness of 2D OIHPs (144 nm), respectively. The charge mobility can be calculated by fitting the I-V curves to the Mott-Gurney law[17, 24] (Supplementary Eq. 7). The derived trap density and charge mobility are given in Supplementary Table 2.

$$J = \frac{9}{8}\varepsilon_0 \varepsilon_r \mu \frac{V^2}{d^3} \qquad (7)$$

Supplementary Note 5. Fluorescence spectra at 80K and their Gaussian fit of six 2D OIHPs

In order to estimate the emission intensity ratio (I_{BE}/I_{NE}) between free exciton state and

self-trapped excited states in six 2D OIHPs, fluorescence peaks from band gap emission (≤ 150 K) were fitted with two or three Gaussians, as shown in Supplementary Figure 8. Here, the blue, green and cyan lines are Gaussian fits to NE1, NE2 and NE3, the magenta line is Gaussian fit to BE. The integrated emission intensity was used to calculate I_{BE}/I_{NE} in each case. It is noting that the simple Gaussian fitting used here should be taken as a semi-quantitative estimate of the I_{BE}/I_{NE} ratios[25,26].

Supplementary Note 6. Spectral fitting of SFG-VS signals

The SFG spectra were fitted using the standard procedure described by Supplementary Eq. 8[27-29].

$$I_{SFG} \propto \left|\chi_{eff}^{(2)}(\omega)\right|^2 = \left|\chi_{NR}^{(2)} + \sum_{v} \frac{A_v}{\omega - \omega_v + i\Gamma_v}\right|^2 \qquad (8)$$

where A_v, ω_v and Γ_v are the amplitude, resonant frequency, and the line width of the vibrational mode (v), respectively. $\chi_{NR}^{(2)}$ is non resonant signal. A_v could be either positive or negative depending on the phase of the vibrational mode. A_v, ω_v and Γ_v can be extracted by fitting the spectrum. We defined the effective peak strength ($\chi_v^{(2)}$) as the fitting strength A_v normalized by Γ_v.

Supplementary Note 7. The possible influence of chain orientation and film thickness on the $\chi_{ss-CH_3}^{(2)}/\chi_{ss-CH_2}^{(2)}$ ratio

There are some concerns that the $\chi_{ss-CH_3}^{(2)}/\chi_{ss-CH_2}^{(2)}$ ratio may be affected by the chain orientation and the film thickness. Theoretically, the ratio of $\chi_{ss-CH_3}^{(2)}/\chi_{ss-CH_2}^{(2)}$ not onlydepends on the fraction of defected chains but also relies on the orientation of the hydrocarbon chain.

The susceptibilities of the symmetric stretch of the methylene group and the methyl symmetric stretch have been given by many publications[29-32](Supplementary Eq. 9-10).

For the symmetric stretch of the methylene group

$$\chi_{ss-CH_2}^{(2)}(\text{yyz}) = \frac{1}{4} N_{CH_2} (\beta_{aac}^{CH_2} + \beta_{bbc}^{CH_2} + 2\beta_{ccc}^{CH_2}) \langle \cos\theta \rangle + \frac{1}{4} N_{CH_2} (\beta_{aac}^{CH_2} + \beta_{bbc}^{CH_2} - 2\beta_{ccc}^{CH_2}) \langle \cos^3\theta \rangle \qquad (9)$$

For the methyl symmetric stretch

$$\chi^{(2)}_{ss-CH_3}(\text{yyz}) = \frac{1}{2} N_{CH_3} \beta^{CH_3}_{ccc} \left[(1+r)\left\langle \cos\theta \right\rangle - (1-r)\left\langle \cos^3\theta \right\rangle \right] \quad (10)$$

where the β are the relevant hyperpolarizability tensor elements, $r = \beta^{CH_3}_{aac} / \beta^{CH_3}_{ccc}$ is the hyperpolarizability ratio. N_{CH_2} and N_{CH_3} are the densities of the respective molecular groups. $\chi^{(2)}(\text{yyz})$ is the component of $\chi^{(2)}$ in a laboratory coordinate system with the z axis pointing along the surface normal and y perpendicular to the plane of incidence. The $\chi^{(2)}_{ss-CH_3}/\chi^{(2)}_{ss-CH_2}$ ratio in the ssp spectra is then given by Supplementary Eq. 11. $B_{ss-CH_3} / B_{ss-CH_2}$ responds the contribution of chain orientation.

$$\frac{\chi^{(2)}_{ss-CH_3}}{\chi^{(2)}_{ss-CH_2}} = \frac{N_{CH_3}}{N_{CH_2}} \frac{\frac{1}{2}\beta^{CH_3}_{ccc}\left[(1+r)\left\langle \cos\theta \right\rangle - (1-r)\left\langle \cos^3\theta \right\rangle \right]}{\frac{1}{4}(\beta^{CH_2}_{aac} + \beta^{CH_2}_{bbc} + 2\beta^{CH_2}_{ccc})\left\langle \cos\theta \right\rangle + \frac{1}{4}(\beta^{CH_2}_{aac} + \beta^{CH_2}_{bbc} - 2\beta^{CH_2}_{ccc})\left\langle \cos^3\theta \right\rangle} = \frac{N_{CH_3}}{N_{CH_2}} \frac{B_{ss-CH_3}}{B_{ss-CH_2}} \quad (11)$$

We assumed a δ-function for θ. For CH_2 group, β_{aac}/β_{ccc} = 1.67, β_{bbc}/β_{ccc} = 0.33 as calculated from the dipole moment and the polarizability derivative of a single C−H bond[33]. For CH_3 group, r =2.3 was experimentally determined by Zhang et al[34]. Based on the bond additivity model $\beta^{CH_2}_{ccc} / \beta^{CH_3}_{ccc}$ =6.8[35,36]. It can be seen that the ratio of $B_{ss-CH_3} / B_{ss-CH_2}$ changes by a factor of 1.39 over the full 90° range of orientation angles. However, if we restrict the considered range of orientation angles from 0° to 30°, this reduces to a factor of 1.076. Actually, previous studies have also demonstrated that a dramatic reduction in the order ratio without introduction of more defects must require a significant variation of the orientation angle[32, 36-38]. In our study, it is found that the average orientation of the terminal methyl groups changes very small for these six 2D OIHPs films because the measured ppp and ssp spectral intensity ratio ($\chi^{(2)}_{ppp}(CH_3,ss)/\chi^{(2)}_{ssp}(CH_3,ss)$ is almost the same (0.9 ± 0.1) (Supplementary Figure 9, 10 and 11). Herein, the ratio of $\chi^{(2)}_{ss-CH_3}/\chi^{(2)}_{ss-CH_2}$ is dominated by the defected chains and not the chain orientation.

To evaluate the influence of the film thickness, we further measure the SFG spectra of the films with the thicknesses of 9.0 nm and 55 nm (Supplementary Figure 10 and 11). It is

found that although the $\chi^{(2)}_{ss-\ CH_3}/\chi^{(2)}_{ss-\ CH_2}$ ratio has a little variation for different thicknesses, the dependence of the $\chi^{(2)}_{ss-\ CH_3}/\chi^{(2)}_{ss-\ CH_2}$ ratio on the alkyl chain length follows similar trend for the films with the same thickness. Furthermore, the surface roughness of the 144 nm-thick films determined by AFM is 2.0±1.0 nm for the films of n=4-8 and 10.0±2.0 nm for the films of n=10-18. There is no correlation between the alkyl chain conformation and the roughness (Supplementary Figure 12).

Supplementary Note 8. Layout of the employed optical pump-Terahertz probe (OPTP) system

Supplementary Figure 14 shows the layout of the employed OPTP system. Femtosecond pulses with 45 fs durations, 800 nm wavelengths were delivered from an amplified Ti: sapphire laser system (Coherent Legend) with a repetition rate of 1 kHz. The laser beam was divided into three portions for the generation and gating of Terahertz (THz) transients, as well as for the optical excitation of the sample. The THz pulse was generated via optical rectification in a [110] ZnTe crystal. The sample was placed at the focus of the THz probe beam and it was photoexcited with a collinear optical pump beam. The pump pulse with wavelength of 400nm was produced with double frequency from a BBO. The spot diameters on the samples for the THz probe beam and the pump beam were about 1.0 mm and4.0 mm, respectively. The pump fluence was varied between16 and 73.6 $\mu J\cdot cm^{-2}$ using neutral density filters. Free-space electro-optic sampling in a second [110] ZnTe crystal was used to coherently detect the THz pulse transmitted through the sample. The optical chopper was placed in either the THz generation path for the detection of the THz electric field waveform or the optical pump path in order to record the photoinduced transmission change. Furthermore, the photoinduced transmission change at the main peak of THz pulse, $\Delta T/T = (T_P-T)/T$, was recorded by varying the optical pump-THz probe delay, in which the delay of optical gating pulse with respect to the THz pulse was fixed at the maximum of the terahertz field. Here T_P and T are the transmissions at THz peak in the case of with and without photoexcitation, respectively. The sign of the pump-probe signal for the measured 2D OIHP films was verified by comparison with the measurement of a standard silicon sample, keeping the same parameter settings of the optical chopper and the lock-in amplifier. All the

experiments were performed under a dry nitrogen purge at room temperature.

Supplementary Note 9. The XRD patterns of six 2D OIHP thin films

The film XRD patterns were obtained on a Philips X'Pert Pro Super diffractometer equipped with graphite monochromatized Cu Kαradiation (λ = 1.54178 Å). Spectra were collected with an angular range of 5° < 2θ < 60° and Δθ = 0.01°. The transmission and reflection spectra were measured with a SolidSpec-3700 UV-vis-NIR spectrophotometer (Shimadzu Corporation, Japan) with blank substrate correction, spectra were collected with a wavelength range of 200 nm ≤ λ≤ 800 nm and Δλ= 1nm.

Fig.4a shows the XRD profiles of six 2D OIHP films. Obviously, these materials have typical (00*l, l*=2, 4, 6, 8, 10 and 12) diffraction peaks, implying that the 2D layered structure of perovskites have been formed[39,40]. We determined the interlayer distance of these 2D OIHP thin films in terms of the positions of (002) diffraction peak (Fig.4a). The peak positions agree well with the values modeled using the software of VESTA[41-43](Supplementary Figure 13 and Supplementary Table 3). It can be seen that the (002) diffraction peaks display a shift toward smaller angles as the organic length increases (from BA_2PbI_4 (n=4) to ODA_2PbI_4 (n=18)). Based on Bragg's law, the interlayer distances are established as 1.36 nm (n=4), 1.66 nm (n=6), 1.83 nm (n=8), 2.08 nm (n=10), 2.38 nm (n=12) and 3.08 nm (n=18), respectively (Fig.4b)).

Supplementary References

1. Milot, R. L., Eperon, G. E., Snaith, H. J., Johnston, M. B. & Herz, L. M. Temperature-dependent charge-carrier dynamics in $CH_3NH_3PbI_3$ perovskite thin films. *Adv. Funct. Mater.* **25**, 6218–6227 (2015).
2. Ponseca, C. S., Savenije, T. J., Abdellah. M., Zheng, K. B., Yartsev. A., Pascher, T., Harlang, T., Chabera, P., Pullerits, T., Stepanov, A., Wolf, J-P. & Sundström, V. Organometal halide perovskite solar cell materials rationalized: ultrafast charge generation, high and microsecond-long balanced mobilities, and slow recombination. *J.Am. Chem. Soc.***136**, 5189–5192 (2014).
3. Valverde-Chavez, D.A., Ponseca, C. S., Stoumpos, C. C., Yartsev, A., Kanatzidis, M. G.,

Sundstrom, V. & Cooke, D. G. Intrinsic femtosecond charge generation dynamics in single crystal $CH_3NH_3PbI_3$. *Energy Environ. Sci.* **8**, 3700–3707 (2015).

4. Wehrenfennig, C., Liu, M. Z., Snaith, H. J., Johnston, M. B. & Herz, L. M. Charge-carrier dynamics in vapour-deposited films of the organolead halide perovskite $CH_3NH_3PbI_{3-x}Cl_x$. *Energy Environ. Sci.* **7**, 2269-2275(2014).
5. Karakus, M., Jensen, S. A ., D'Angelo, F., Turchinovich, D., Bonn, M. & Canovas, E. Phonon–electron scattering limits free charge mobility in methylammonium lead iodide perovskites. *J. Phys. Chem. Lett.* **6**, 4991-4996 (2015).
6. Rehman, W., Milot, R. L., Eperon, G. E., Wehrenfennig, C., Boland, J. L., Snaith, H. J., Johnston, M. B. & Herz, L. M. Charge-carrier dynamics and mobilities in formamidinium lead mixed-halide perovskites. *Adv. Mater.* **27**, 7938–7944 (2015).
7. Rehman, W., McMeekin, D. P., Patel, J. B., Milot, R. L., Johnston, M. B., Snaith, H. J. & Herz, L. M. Photovoltaic mixed-cation lead mixed-halide perovskites: links between crystallinity, photo-stability and electronic properties. *Energy Environ. Sci.* **10**, 361-369 (2017).
8. McMeekin, D. P., Sadoughi, G., Rehman, W., Eperon, G. E., Saliba, M., Horantner, M. T., Haghighirad, A., Sakai, N., Korte, L., Rech, B., Johnston, M. B., Herz, L. M. & Snaith, H. J. A mixed-cation lead mixed-halide perovskite absorber for tandem solar cells. *Science* **351**, 151-155 (2016).
9. McMeekin, D. P., Wang, Z. P., Rehman, W., Pulvirenti, F., Patel, J. B., Noel, N. K., Johnston, M. B., Marder, S. R., Herz, L. M. & Snaith, H. J. Crystallization kinetics and morphology control of formamidinium–cesium mixed-cation lead mixed-halide perovskite via tunability of the colloidal precursor solution. *Adv. Mater.* **29**, 1607039 (2017).
10. Noel, N. K., Stranks, S. D., Abate, A., Wehrenfennig, C., Guarnera, S., Haghighirad, A. A., Sadhanala, A., Eperon, G. E., Pathak, S. K., Johnston, M. B., Petrozza, A., Herz, L. M.& Snaith, H. J. Lead-free organic–inorganic tin halide perovskites for photovoltaic applications. *Energy Environ. Sci.* **7**, 3061-3068 (2014).
11. Milot, R. L., Eperon, G. E., Green, T., Snaith, H. J., Johnston, M. B. & Herz, L. M. Radiative monomolecular recombination boosts amplified spontaneous emission in

$HC(NH_2)_2SnI_3$perovskite films. *J. Phys. Chem. Lett.* **7**, 4178-4184 (2016).

12. Eperon, G. E., Leijtens, T., Bush, K. A., Prasanna, R., Green, T., Wang, J. T. W., McMeekin, D. P., Volonakis, G., Milot, R. L., May, R., Palmstrom, A., Slotcavage, D. J., Belisle, R. A., Patel, J. B., Parrott, E. S., Sutton, R. J., Ma, W., Moghadam, F., Conings, B., Babayigit, A., Boyen, H. G., Bent, S., Giustino, F., Herz, L. M., Johnston, M. B., McGehee, M. D. & Snaith, H. J. Perovskite-perovskite tandem photovoltaics with optimized band gaps. *Science* **354**, 861-865 (2016).
13. Milot, R. L., Sutton, R. J., Eperon, G. E., Haghighirad, A. A., Hardigree, J. M., Miranda, L., Snaith, H. J., Johnston, M. B. & Herz, L. M. Charge-carrier dynamics in 2D hybrid metal–halide perovskites. *Nano Lett.* **16**, 7001–7007 (2016).
14. Buizza, L. R. V., Crothers, T. W., Wang, Z. P., Patel, J. B., Milot, R. L., Snaith, H. J., Johnston, M. B. & Herz, L. M. Charge-carrier dynamics, mobilities, and diffusion lengths of 2D–3D hybrid butylammonium–cesium–formamidinium lead halide perovskites. *Adv. Funct. Mater.***29**, 1902656 (2019).
15. Sanehira, E. M., Marshall, A. R., Christians, J. A., Harvey, S. P., Ciesielski, P. N., Wheeler, L. M., Schulz, P., Lin, L. Y., Beard, M. C. & Luther, J. M. Enhanced mobility $CsPbI_3$ quantum dot arrays for record-efficiency, high-voltage photovoltaic cells. *Sci. Adv.***3**, eaao4204 (2017).
16. Johnston, M. B. & Herz, L. M. Hybrid perovskites for photovoltaics: Charge-carrier recombination, diffusion, and radiative Efficiencies. *Acc. Chem. Res.* **49**, 146-154 (2016).
17. Yu, M. T., Yi, C., Wang, N. N., Zhang, L. D., Zou, R. M., Tong, Y. F., Chen, H., Cao, Y., He, Y. R., Wang, Y., Xu, M. M., Liu, Y., Jin, Y. Z., Huang, W. & Wang, J. P. Control of barrier width in perovskite multiple quantum wells for high performance green light–emitting diodes. *Adv. Optical Mater.* **7**, 1801575 (2019).
18. Hong, X., Ishihara, T. & Nurmikko, A. V. Dielectric confinement effect on excitons in PbI4-based layered semiconductors. *Phys. Rev. B* **45**, 6961−6964 (1992).
19. Shen, Y.R. *The principles of nonlinear optics.* Wiley, New York, **1984**.
20. Lambert, A.G., Davies, P.B. & Neivandt, D.J. Implementing the theory of sum frequency generation vibrational spectroscopy: a Tutorial Review. *Appl. Spectrosc. Rev.* **40**, 103-145 (2005).

21. Wang, H. F., Gan, W., Lu, R., Rao, Y. &Wu, B. H. Quantitative spectral and orientational analysis in surface sum frequency generation vibrational spectroscopy (SFG-VS). *Int. Rev. Phys. Chem.* **24,** 191−256 (2005).
22. Zhuang, X. W., Miranda, P. B. & Shen, Y. R. Mapping molecular orientation and conformation at interfaces by surface nonlinear optics, *Phys. Rev. B* **59,** 12632 (1999).
23. Nguyen, Nguyen, K. T. &Nguyen, A. V. New evidence of head-to-tail complex Formation of SDS−DOH mixtures adsorbed at the air−water interface as revealed by vibrational sum frequency generation spectroscopy and isotope labelling. *Langmuir* **35,** 4825−4833 (2019).
24. Beier-Hannweg, A., Firla, D. & Hasselbrink, E. Order and melting stability of calcium arachidate Langmuir-Blodgett monolayers prepared at different pH. *Thin Solid Films* **642,** 1–7 (2017).
25. Wei, X., Hong, S. C., Zhuang, X. W., Goto, T. & Shen, Y. R. Nonlinear optical studies of liquid crystal alignment on a rubbed polyvinyl alcohol surface. *Phys. Rev. E* **62,** 5160−5172 (2000).
26. Zhang, D., Gutow, J. &Eisenthal, K. B. Vibrational-spectra, orientations, and phase-transitions in long-chain amphiphiles at the air-water-interface - probing the head and tail groups by sum-frequency generation. *J. Phys. Chem.* **98,** 13729−13734 (1994).
27. Hirose, C., Akamatsu, N. &Domen, K. Formulas for the analysis of surface sum-frequency generation spectrum by CH stretching modes of methyl and methylene groups. *J. Chem. Phys.***96,** 997–1004 (1992).
28. Bordenyuk, A. N., Weeraman, C., Yatawara, A., Jayathilake, H. D., Stiopkin, I., Liu, Y. & Benderskii, A. V. Vibrational sum frequency generation spectroscopy of dodecanethiol on metal nanoparticles. *J. Phys. Chem. C* **111,** 8925-8933 (2007).
29. Zhang, H., Li, F. J., Xiao, Q. B. &Lin, H. Z. Conformation of capping ligands on nanoplates: Facet-edge induced disorder and self-assembly-related ordering revealed by sum frequency generation spectroscopy. *J. Phys. Chem. Lett.* **6,** 2170−2176 (2015).
30. Schleeger, M., Nagata, Y. &Bonn, M. Quantifying surfactant alkyl chain orientation and conformational order from sum frequency generation spectra of CH modes at the surfactant−water interface. *J. Phys. Chem. Lett.* **5,** 3737−3741 (2014).

31. Cinquanta, E., Meggiolaro, D., Motti, S. G., Gandini, M., Alcocer, M. J., Akkerman, Q. A., Vozzi, C., Manna, L., De Angelis, F., Petrozza, A. & Stagira, S. Ultrafast THz probe of photoinduced polarons in lead-halide perovskites. *Phys. Rev. Lett.* **122**, 166601 (2019).
32. Yettapu, G. R., Talukdar, D., Sarkar, S., Swarnkar, A., Nag, A., Ghosh, P. & Mandal, P. Terahertz conductivity within colloidal $CsPbBr_3$ perovskite nanocrystals: Remarkably high carrier mobilities and large diffusion lengths. *Nano Lett.* **16**, 4838-4848 (2016).
33. Kar, S., Su, Y., Nair, R. R. & Sood, A. K. Probing photoexcited carriers in a few-layer MoS_2 laminate by time-resolved optical pump-terahertz probe spectroscopy. *ACS Nano* **9**, 12004-12010 (2015).
34. Xing, X., Zhao, L. T., Zhang, Z., Liu, X. K., Zhang, K. L., Yu, Y., Lin, X., Chen, H. Y., Chen, J. Q., Jin, Z. M., Xu, J. H. & Ma, G. H. Role of photoinduced exciton in the transient terahertz conductivity of few-layer WS_2 laminate. *J. Phys. Chem. C* **121**, 20451-20457 (2017).
35. Gao, B., Hartland, G., Fang, T., Kelly, M., Jena, D., Xing, H. G. & Huang, L. B. Studies of intrinsic hot phonon dynamics in suspended graphene by transient absorption microscopy. *Nano Lett.* **11**, 3184-3189 (2011).
36. Joglekar, S. G., Hammig, M. D. &Guo, L. J. High-energy photon spectroscopy using all solution-processed heterojunctioned surface-modified perovskite single crystals. *ACS Appl. Mater. Interfaces* **11,** 33399-33408 (2019).
37. Wu, X. X., Trinh, M. T., Niesner, D., Zhu, H. M., Norman, Z., Owen, J. S., Yaffe, O., Kudisch, B. J. & Zhu, X. Y. Trap states in lead iodide perovskites. *J. Am. Chem. Soc.* **137**, 2089-2096 (2015).
38. Smith, M. D., Jaffe, A., Dohner, E. R., Lindenberg, A. M. & Karunadasa, H. I. Structural origins of broadband emission from layered Pb-Br hybrid perovskites. *Chem. Sci.* **8**, 4497-4504 (2017).
39. Gan, L., Li, J., Fang, Z. S., He, H. P. & Ye, Z. Z. Effects of organic cation length on exciton recombination in two-dimensional layered lead iodide hybrid perovskite crystals. *J. Phys. Chem. Lett.* **8,** 5177-5183 (2017).
40. Yang, S., Niu, W., Wang, A. L., Fan, Z., Chen, B., Tan, C., Lu, Q. & Zhang, H. Ultrathin two-dimensional organic-inorganic hybrid perovskite nanosheets with bright, tunable

photoluminescence and high stability. *Angew. Chem., Int. Ed.* **56**, 4252 (2017).

41. Billing, D. G. & Lemmerer, A. Synthesis, characterization and phase transitions in the inorganic–organic layered perovskite-type hybrids [$C_nH_{2n+1}NH_3)_2PbI_4$], *n*=4, 5 and 6. *Acta Crystallogr. B* **63**, 735–747 (2007).
42. Lemmerer, A. & Billing, D. G. Synthesis, characterization and phasetransitions of the inorganic–organic layered perovskite-type hybrids [$(C_nH_{2n+1}NH_3)_2PbI_4$], *n*=7, 8, 9 and 10. *Dalton Trans.* **41**, 1146–1157 (2012).
43. Billing, D. G. & Lemmerer, A. Synthesis, characterization and phase transitions of the inorganic-organic layered perovskite-type hybrids [$(C_nH_{2n+1}NH_3)_2PbI_4$] (n = 12, 14, 16 and 18). *New. J. Chem.* **32,** 1736–1746 (2008).
